# Behavioural Adjustment in Response to Increased Predation Risk: A Study in Three Duck Species

**DOI:** 10.1371/journal.pone.0018977

**Published:** 2011-04-20

**Authors:** Cédric Zimmer, Mathieu Boos, Frédéric Bertrand, Jean-Patrice Robin, Odile Petit

**Affiliations:** 1 Département Ecologie, Physiologie et Ethologie, Institut Pluridisciplinaire Hubert Curien, Université de Strasbourg, CNRS, Strasbourg, France; 2 Naturaconst@ Research Agency in Applied Ecology, Wilshausen, France; 3 Institut de Recherche en Mathématique Avancée, Université de Strasbourg, CNRS, Strasbourg, France; University of Western Ontario, Canada

## Abstract

Predation directly triggers behavioural decisions designed to increase immediate survival. However, these behavioural modifications can have long term costs. There is therefore a trade-off between antipredator behaviours and other activities. This trade-off is generally considered between vigilance and only one other behaviour, thus neglecting potential compensations. In this study, we considered the effect of an increase in predation risk on the diurnal time-budget of three captive duck species during the wintering period. We artificially increased predation risk by disturbing two groups of 14 mallard and teals at different frequencies, and one group of 14 tufted ducks with a radio-controlled stressor. We recorded foraging, vigilance, preening and sleeping durations the week before, during and after disturbance sessions. Disturbed groups were compared to an undisturbed control group. We showed that in all three species, the increase in predation risk resulted in a decrease in foraging and preening and led to an increase in sleeping. It is worth noting that contrary to common observations, vigilance did not increase. However, ducks are known to be vigilant while sleeping. This complex behavioural adjustment therefore seems to be optimal as it may allow ducks to reduce their predation risk. Our results highlight the fact that it is necessary to encompass the whole individual time-budget when studying behavioural modifications under predation risk. Finally, we propose that studies of behavioural time-budget changes under predation risk should be included in the more general framework of the starvation-predation risk trade-off.

## Introduction

All animals potentially face predation events. Indeed, predation clearly appears to be an important factor for the selection of morphological and behavioural characters [1], [2]. In response to an increase in predation risk, animals usually alter their behaviour to avoid being caught by predators or to keep them away [1], [2], [3], [4], [5], [6]. Although these behavioural decisions increase immediate survival, they could have potential costs such as decreased access to food and less probability to find mates, or lead to shift to possible less suitable habitats. Consequently, the increase of immediate survival could have long-term effects by decreasing growth rate or reproductive output [2], [4], [7], [8]. Thus, a useless or extreme response can also be a waste of time or energy for prey [9], [10]. There is therefore a trade-off between antipredator behaviours, which increase immediate survival, and other behavioural activities [2], [5].

The majority of previous studies on the effects of predation risk on behavioural modifications have focused solely on the trade-off between a single antipredator behaviour, usually vigilance, and one other activity such as foraging, sleeping, drinking, mating or parental investment [1], [2], [7], [11], [12], [13]. These studies show an increase in the time allocated to antipredator behaviours at the expense of time allocated to other behaviours. However, because behaviours are not independent from each other, potential more complex compensations may exist [5], [14]. Moreover, time allocation for different behaviours may depend on other ecological constraints such as starvation risk, and thus may not be a response to predation risk alone [4], [5]. The measurement of time-budget in individuals therefore appears necessary in order to take into account the different constraints at work and understand optimal behavioural adjustment, if any [5], [14].

Birds are a useful model when studying how predation risk affects behaviour, as vigilance can easily be distinguished from other activities [2]. Moreover, non-lethal effects of predation risk appear to be particularly present in the avian lineage [4]. Among birds, waterfowl - and especially ducks - are particularly exposed to predation, since they live in open water and terrestrial sites without cover [15], [16], [17]. Individuals can be attacked by terrestrial predators but also by raptors or even gulls. Ducks can dive in response to these attacks, but most often they fly away to reach cover (high vegetation) or another pond [18], [19], [20], [21], [22]. Moreover, the fact that vigilance for aerial/terrestrial predators is impossible for ducks when foraging with heads under water makes them particularly suitable models for studying the starvation-predation trade-off. Furthermore, ducks species differ in their ecology, body size or response to disturbance [23], [24]. As far as we know, however, little information is available about how these differences between ducks species may affect their behavioural responses to predation risk. Here, we used a comparative approach with the study of three representative species of *Anatidae*: the mallard (*Anas platyrhynchos*), the common teal (*Anas crecca*, hereafter referred to as “teal”), and the tufted duck (*Aythya fuligula*). Mallards and teals are among the largest and smallest duck species, respectively. Moreover, mallards and teals differ in their ecology compare to tufted ducks which are diving ducks and rely more on protein food [25]. In order to study the relationships between predation risk and behaviour in these species we experimentally increased the risk of predation by exposing birds to a chronic disturbance in which we simulated attacks from a predator. Experiments were conducted under controlled conditions with the same protocol to ensure that the context was the same for all individuals of the three species [3]. We encompassed the time-budget for each duck in order to investigate compensations between different behaviours. We therefore predicted that in the given situation, ducks would reduce behaviours that enhance predation risk while maintaining those behaviours that could lower starvation risk [4], [26]. Because, larger bird species have higher mechanical constraints to initiate flight [27] but have higher body fuel storage capabilities, i.e higher fasting endurance, we expect that vigilance would be more reduced and energy-saving behaviours more enhanced in small than in large body sized species (teals *versus* tufted ducks *versus* mallards). Therefore, we will consider expected behavioural changes under predation risk in the more general framework of the starvation-predation risk trade-off.

## Results

### General effects

The only significant effect of the sex on behaviours was recorded in mallards. In this species, foraging time was higher in females (333±35 s) than in males (187±17 s) (*F*_1,28_ = 4.94, *P* = 0.037), and vigilance duration was lower in females (288±17 s) than in males (393±19 s) (*F*_1,44_ = 22.40, *P*<0.0001). Instead, no significant behavioural modification was observed between weeks among the individuals of the three control groups for all sessions and between sessions for all the behaviours in each group of the three species (*P*>0.05).

### Mallards

**Figure 1:**
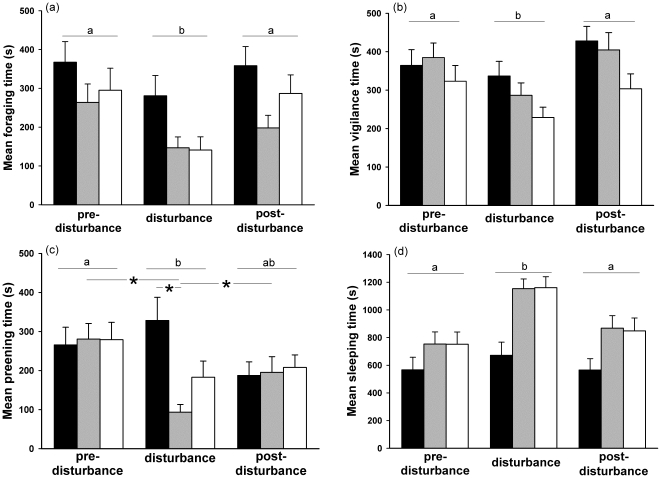
Time budget in mallards. (a) Foraging, (b) vigilance, (c) preening and (d) sleeping behaviours in the control group (black bar), group 1 (grey bar) and group 2 (white bar) for each observation week. Letters indicate significant differences between weeks. *Indicates significant differences between weeks for a group or between two groups.

Generally, foraging time was significantly different in the three groups (*F*_2,26_ = 6.57, *P* = 0.0049), being higher for ducks of the CG (333±30 s) than for ducks of the two disturbed groups (G1: 203±21 s; G2: 241±28 s) (*t*_19_<−2.22, *P*<0.039). Foraging was also different over the three weeks of observation (*F*_2,293_ = 9.88, *P*<0.0001) with lower durations during the week of disturbance (187±30 s) compared to the pre- (309±31 s) and post-disturbance weeks (281±29 s) (*t*_284_>3.31; *P*<0.003) (Figure 1a).

Vigilance time differed significantly among groups (*F*_2,44_ = 8.35, *P* = 0.0008). It was lower for ducks of G2 (285±21 s) than for ducks of G1 (360±23 s) and of the CG (376±23 s) (*t*_44_<−3.17, *P*<0.0076). A difference in vigilance duration was observed between weeks (*F*_2,316_ = 4.88, *P* = 0.0081) with higher values during the weeks preceding (357±23 s) and following (379±24 s) the disturbance week (283±19 s) (*t*_312_>2.74, *P*<0.018) (Figure 1b).

Preening duration differed significantly according to weeks (*F*_2,317_ = 3.83, *P* = 0.023), with higher values recorded before disturbance than during disturbance (251±26 s vs 161±34 s; *t*_316_ = 2.77, *P* = 0.016). Moreover, less preening was observed for ducks of G1 (166±20 s) than for either of ducks of the other groups (G2: 223±23 s; CG: 266±28 s, *t*_44_<−2.90, *P*<0.017). Lastly, the interaction between weeks and groups was significant (*F*_4,352_ = 4.98, *P* = 0.0006). During disturbance week in G1, less preening occurred than during the two other weeks (*t*_316_<−3.25, *P*<0.034) and less preening was observed than in the CG (*t*_143_ = 5.50, *P*<0.0001) (Figure 1c).

Sleeping duration was lower for ducks of the CG (601±52 s) than for ducks of disturbed groups (G1: 925±50 s; G2: 920±52 s) (*t*_49_>4.02, *P*<0.0006). There was also a significant difference in sleeping duration between weeks (*F*_2,306_ = 8.50, *P* = 0.0003), with higher values during the week of disturbance (996±55 s) than in the other weeks (before disturbance: 691±53 s; after disturbance: 761±47 s) (*t*_285_>2.29, *P*<0.05) (Figure 1d).

**Figure 2:**
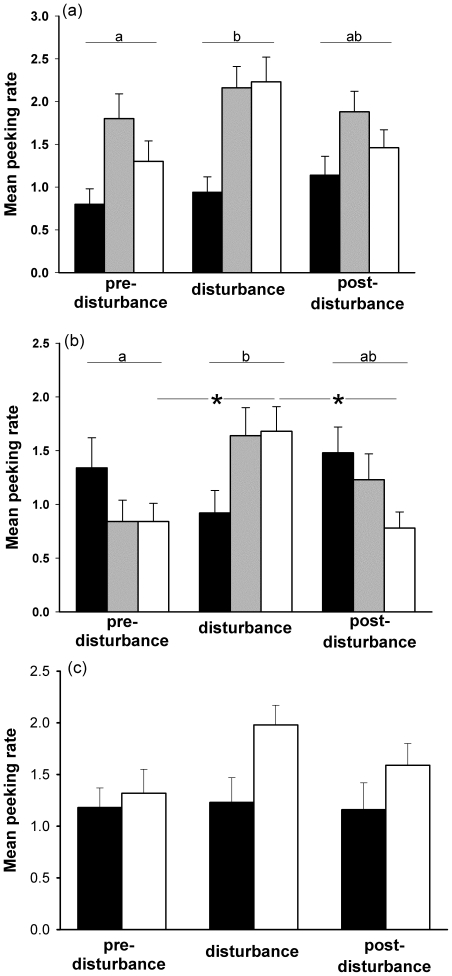
Peeking rate in mallards (a), in teals (b) and in tufted ducks (c) for the control group (black bar), group 1 (grey bar) and group 2 (white bar) for each observation week. Letters indicate significant differences between weeks. *Indicates significant differences between weeks for a group or between two groups.

The number of peeks during sleeping differed significantly between ducks of the three groups (*F*_2,34_ = 8.40, *P* = 0.001). Peeking rate was higher for ducks of G1 (1.95±0.15) and of G2 (1.67±0.15) than for ducks of the CG (0.96±0.11) (*t*_24_>3.46, *P*<0.004). Finally, the peek number was different between the three weeks of observation (*F*_2,281_ = 4.28, *P* = 0.015) with lower frequencies before disturbance (1.30±0.15) than during disturbance (1.78±0.15) (*t*_285_ = −2.90, *P* = 0.011) (Figure 2a).

### Teals

**Figure 3:**
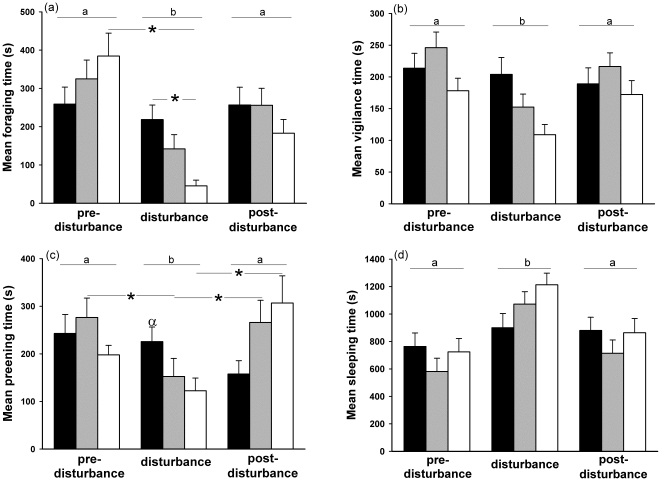
Time budget in teals. (a) Foraging, (b) vigilance, (c) preening and (d) sleeping behaviours for the week before the disturbance, the week of disturbance and the week after the disturbance for the control group (black bar), group 1 (grey bar) and group 2 (white bar) for each observation week. Letters indicate significant differences between weeks. *Indicates significant differences between weeks for a group or between two groups. α indicates that behaviour duration was significantly different for the control group compared to the two disturbed groups during the week of disturbance.

Foraging duration differed according to observation weeks (*F*_2,285_ = 14.74, *P*<0.0001). Foraging duration was approximately 2-fold lower during the disturbance week (135±19 s) than during the preceding (323±29 s) and following weeks (232±24 s) (*t*_66_<−3.33, *P*<0.004). Moreover, the interaction between weeks and groups was significant (*F*_4,320_ = 2.95, *P* = 0.0259). In G2, ducks foraged less during the week of disturbance than during the week before and foraged also less than ducks of the CG (*t*_81_<−2.62, *P*<0.05) (Figure 3a).

Vigilance duration differed significantly between ducks of the three groups (*F*_2,44_ = 6.93, *P* = 0.0024), being lower for ducks of G2 (152±11 s) than for ducks of G1 (204±13 s) and of the CG (202±14 s) (*t*_44_<−3.19, *P*<0.007). Vigilance time was also significantly different between the three weeks (*F*_2,312_ = 7.08, *P* = 0.001). It was higher before (212±13 s) and after (193±13 s) than during the disturbance (155±13 s) (*t*_315_>2.78, *P*<0.016) (Figure 3b).

Preening duration was different according to weeks (*F*_2,290_ = 8.68, *P* = 0.0002), being lower during the week of disturbance (167±19 s) compared to other weeks (before: 239±21 s; after: 244±26 s) (*t*_305_<−3.32, *P*<0.0029). Interaction between weeks and groups was significant (*F*_4,325_ = 5.79, *P* = 0.0002). For ducks of G2, preening time was lower during the week of disturbance than the week after (*t*_305_ = −3.73, *P* = 0.007) (Figure 2c). For ducks of G1, the decrease was more pronounced since it was lower during disturbance than both before and after (*t*_305_<−3.60, *P*<0.01) (Figure 3c). Lastly, during the disturbance week, preening duration was higher for ducks of the CG than for ducks of G1 and of G2 (*t*_123_>3.21, *P*<0.032) (Figure 3c).

Sleeping behaviour only varied significantly according to weeks (*F*_2,287_ = 10.79, *P*<0.0001). Sleeping duration was higher during the disturbance week (1062±54 s) compared to the weeks before (690±56 s) and after (820±57 s) the disturbance (*t*_273_>2.92, *P*<0.01) (Figure 3d).

Peeking rate differed significantly between the three weeks of observation (*F*_2,264_ = 4.40, *P* = 0.013) with lower frequencies during the week preceding disturbance (1.01±0.13) than during the week of disturbance (1.42±0.13) (*t*_229_ = −2.66, *P* = 0.022). Interaction between weeks and groups was also significant (*F*_4,290_ = 5.28, *P* = 0.0004). For ducks of G2, peeking rate was higher during the week of disturbance (1.68±0.23) compared to the preceding (0.84±0.17) and following (0.78±0.15) weeks (*t*_173_>3.08, *P*<0.05) (Figure 2b).

### Tufted ducks

**Figure 4:**
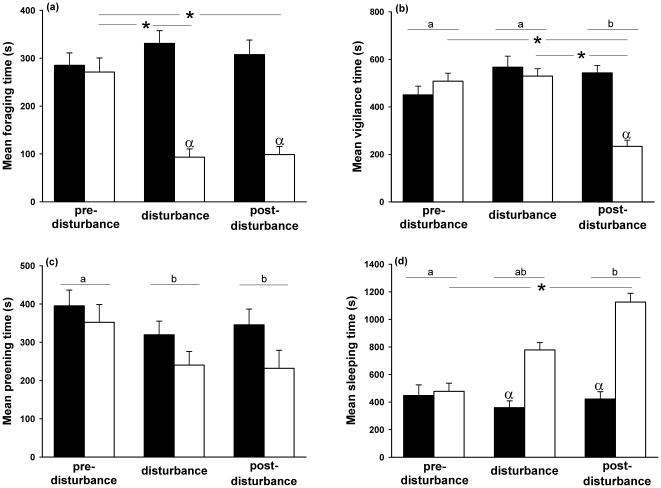
Time budget in tufted ducks. (a) Foraging, (b) vigilance, (c) preening and (d) sleeping behaviours for the week before the disturbance, the week of disturbance and the week after the disturbance for the control group (black bar) and group 2 (white bar) for each observation week. Letters indicated significant differences between weeks. *Indicates significant differences between weeks for a group or between two groups. α indicates that behaviour duration was significantly different for the control group compared to the disturbed group during the week of disturbance and the week after disturbance.

Foraging duration was significantly different between ducks of the control group (308±26 s) and of the disturbed group (154±19 s) (*F*_1,33_ = 22.56, *P*<0.0001). Interaction between weeks and groups was also significant (*F*_2,188_ = 5.99, *P* = 0.005). In G2, ducks foraged less during and after the disturbance than before it (*t*_42_<−3.31, *P*<0.022). Moreover, ducks of G2 foraged less than those of the CG over these last two weeks of observation (*t*_45_>3.99, *P*<0.003) (Figure 4a).

Vigilance duration was significantly higher for ducks of the control group (520±25 s) than for ducks of the disturbed group (424±27 s) (*F*_1,39_ = 13.19, *P* = 0.0008). The interaction between weeks and groups was significant (*F*_2,194_ = 15.76, *P*<0.0001). In G2, ducks were less vigilant during the week following disturbance than in the two previous weeks (*t*_187_<−6.22, *P*<0.0001). Furthermore, after disturbance, vigilance was higher for ducks of the control group than for ducks of the disturbed one (*t*_103_ = 5.82, *P*<0.0001) (Figure 4b).

Preening duration was significantly lower for ducks of the disturbed group (275±31 s) compared to those of the control group (354±28 s) (*F*_1,32_ = 10.52, *P* = 0.003) and also differed significantly between weeks (*F*_2,191_ = 4.72, *P* = 0.01). Preening time was higher during the week before disturbance (374±31 s) than during the two other weeks (disturbance: 280±25 s; after: 289±32 s) (*t*_190_>2.46, *P*<0.04) (Figure 4c).

Sleeping duration was higher for ducks of the disturbed group (794±52 s) compared to ducks of the control group (410±43 s) (*F*_1,27_ = 18.65, *P* = 0.0002). Moreover, the interaction between weeks and groups was significant (*F*_2,181_ = 4.96, *P* = 0.008). In G2, sleeping duration was lower before disturbance than after (*t*_155_ = −4.21, *P* = 0.0006). In addition, ducks of G2 slept more than those of the CG (*t*_68_>4.06, *P*<0.001) throughout the weeks during and after disturbance (Figure 4c).

Peeking rate was higher for ducks of the disturbed group (1.63±0.12) than for ducks of the control group (1.19±0.13) (*F*_1,31_ = 4.33, *P* = 0.046) (Figure 2c).

## Discussion

We show here that an increased disturbance mimicking an increased [1] predation risk consistently affected time-budget in ducks through decreasing preening, vigilance and foraging, and also resulted in a concomitant increase of sleep duration. These adjustments in diurnal time-budget were approximately equivalent in the three species and were not simply an increase in vigilance at the expense of other behaviours.

Before any disturbance, tufted ducks allocated less time to sleeping and more time to vigilance and preening than mallards and teals. Globally, these time-budgets correspond to those described in the wild (see [28]) for all three species. However, we noticed a higher amount of time allocated to sleeping at the expense of foraging, probably because ducks in our study had *ad libitum* access to food, in a familiar environment. Mallards and teals responded to the increase in predation risk by globally exhibiting the same behavioural adjustments, namely an increase of sleeping duration at the expense of other behaviours such as foraging and vigilance. Similarly, sleeping time also increased in tufted ducks although vigilance in this species was maintained at the same level. In the three species, foraging was the behaviour seen to decrease the most. We observed different adjustments between the two disturbed groups in mallards and teals. It is worth noting that vigilance duration was lower for ducks of G2 (highly disturbed group) during the week of disturbance, whereas the decrease in preening duration was highest for ducks of G1 (moderately disturbed group). One explanation could be that more time was allocated to plumage maintenance at the expense of vigilance in response to higher plumage disorder in highly disturbed ducks of G2. Indeed, plumage maintenance is essential to fly and for bird's survival [29]. The week following disturbance, mallards and teals almost returned to their initial state. Therefore, their behavioural adjustment seemed to be transitional and a direct response to the increase in predation risk. Conversely, this direct response was less marked in tufted ducks but behavioural adjustments persisted after the risk had ceased. Actually, sleeping first increased at the expense of foraging during the disturbance week and continued to increase at the expense of vigilance during the post-disturbance week. These adjustments could be related to nutritional constraints. Indeed, during the first days of the disturbance week, food intake is reduced in disturbed groups whatever the duck species ([30] Zimmer, unpublished data). Then, from the last days of disturbance to the end of the post-disturbance week, food intake increased. Overall this phenomenon was delayed in disturbed tufted ducks, especially for vegetal items (standard commercial food) compared to the protein rich food supplement (Zimmer, unpublished data). Indeed, tufted ducks are heavily dependent on protein food [25], [31], which metabolism involves different energetic benefits and costs [32]. From our data, this selective foraging seemingly remains a priority under high predation risk situations for this species. Then, an energetic imbalance could explain the time discrepancy in behaviour adjustments between this species and granivorous ones. However to answer such assumption, we need to further investigate the whole interaction between behaviour changes and energetic adaptations, especially the ones concerning the nutritional balance intake.

As a whole, we found that general activity was reduced in disturbed groups of ducks during increased predation risk events, as previously observed in other species (reviewed in [2], [7]). Indeed, the decreasing of activity reduces the probability of being detected or encountered by a predator, therefore limiting the risk of predation [2], [7]. Moreover, under a high predation risk, antipredator behaviours such as an increase in vigilance in order to reduce the risk of predation [1] seem to be the most convenient adaptation. This type of response, associated with decreased or frequently interrupted sleep, has been widely described in different free-living bird species [1], [2], [26], [33], [34], [35], [36]. However, the time assigned to vigilance behaviours cannot be allocated to other activities and, in fact, a trade-off between antipredator and other behaviours has already been evidenced in animals [2], [5]. Paradoxically, we found that the time allocated to vigilance did not increase whereas sleep increased in response to disturbance in our three species. To our knowledge, one previous studies led to similar results, rainbow trout (*Oncorhynchus mykiss*) frequently exposed to high risk situations displayed a lower level of antipredator behaviour (i.e. decreased vigilance) compared to those infrequently exposed to risk [6].

Several arguments might clarify that the above adjustments could in fact be beneficial for bird survival. We can suppose that sleep allows energy saving [37], [38]. It could therefore be part of an adequate adaptation, since disturbance led to great number of flights demanding high amounts of energy [39] which our ducks did not compensate with increased food intake despite *ad libitum* access over the 24 hours ([30] Zimmer, unpublished data). However, as sleeping animals are relatively unresponsive and unaware of their proximate environment [12] they are also considered highly vulnerable to predation [12], [40], [41]. Nevertheless, according to the immobilization hypothesis [42], sleep could have a protective role since motionless animals are less detectable. Moreover, birds and especially ducks exhibit vigilant sleep, i.e. alternation between periods of eye closure and peeks that allow birds to scan their environment to detect predators [37], [40]. Indeed, in all three species the peeking rate was higher for ducks of disturbed than for those of control groups, particularly during the week of disturbance (Figure 2). A similar increase in vigilant sleep under high predation risk has been demonstrated in green-winged teals (*Anas crecca crecca*) and in gadwall (*Anas strepera*) [26], [35] or in mallards sleeping in high risk situations [40], [43], [44]. To conclude, since ducks may be vigilant while sleeping, it is conceivable that the increase in sleep and the concomitant decrease in vigilance we observed could be an adequate response allowing both a decrease in predation risk and the sparing of energy.

In response to disturbance, foraging duration also decreased in all three species. This result is typical of high predation risk situations because it is assumed that a trade-off exists between antipredator behaviours and foraging [1], [2], [4], [45]. In accordance with this trade-off, we observed that foraging duration decreased approximately twice as much as vigilance (Figures 1, 3, and 4). This indicates that ducks give priority to antipredator behaviours in comparison to foraging duration. Moreover, such a decrease in foraging activity reduces vulnerability to predation [2], [7], [8], [46], [47]. In fact we show that despite *ad libitum* provision of food throughout both day and night, this decreased foraging time observed during daylight was accompanied by a spontaneous decrease in the total food intake (up to 70%) leading to a decrease in body mass (between 8 and 15%) and wing loading in all three duck species ([30] Zimmer, unpublished data). The result recorded in these 3 nocturnal feeder species [48], [49], [50], [51] preclude any large foraging compensation during the night. Such adjustments should enhance flying capabilities and again decrease predation risk by improving escape performances: low wing loading increases take-off angle as well as speed and aerial manoeuvrability [27], [52], [53]. Moreover, it has been suggested that when birds decrease their body mass in response to predation risk, individual vigilance contributes less to survival [14]. Our results confirmed this assumption.

Our study revealed that diurnal time-budget adjustments in ducks are rather complex. First, we observed the typical decrease in the duration of feeding behaviour in response to an increase in predation risk. However counter-intuitive it may be, we also observed an increase in sleep duration while no increase in vigilance duration could be evidenced. This time-budget adjustment nevertheless appears to be a global strategy that allows ducks to reduce the risk of predation. In accordance with Lind and Cresswell [5], our methodological approach indicates that it seems necessary to take into account the individual's time-budget when studying the impact of predation on behaviour in order to encompass potential compensations. On the contrary, most studies consider trade-offs between just two specific behaviours, for example the trade-off between foraging and antipredator behaviour [8], [11], [54], [55], [56]. Indeed, as we highlighted, measuring the effects of predation risk on a single behaviour can neglect other behavioural compensations and lead to flawed conclusions [5].

We should also take into account that by affecting foraging behaviour, predation risk can consequently affect starvation risk [4]. Indeed, despite an *ad libitum* access to food, the risk of starvation of ducks increased due to the decrease in food intake and also body mass and energy reserves ([30] Zimmer, unpublished data). On the other hand, the observed decrease in vigilance and preening together with the increase in sleep duration may enable ducks to save energy and thus compensate for the risk of starvation. Hence, behavioural decisions may also be influenced by starvation risk [4], [5], [57]. The behavioural adjustments observed in our study adequately fit the starvation-predation risk trade-off. Indeed, in a context of high predation risk, birds should decrease their body mass [30], [47], [58]. Therefore, high body mass requires greater foraging time and metabolic demands and impairs flight capabilities, negative factors that would increase predation risk. However, birds should maintain sufficient body reserves to anticipate fluctuations in food availability [47], [58]. Therefore, it seems that besides physiological and energetic adjustments, behavioural adjustments could be a useful tool to understand response to the starvation-predation risk trade-off in animals.

To conclude, this experimental study of three duck species showed that time-budget adjustments were far from simple. It therefore appears necessary to take into account the individual's time-budget rather than to focus simply on one or two behaviours when studying the effects of predation risk or more generally of disturbance on behaviour. Hence, we propose to integrate behavioural changes occurring in response to variation of predation risk within the context of the starvation-predation risk trade-off. Indeed, we obtained the same behavioural mechanisms to adapt to an increase in predation risk for the three species. These processes are probably independent of the ecology and of size-specific flight mechanic differences between duck species. This provides arguments for the generalization of the starvation-predation risk trade-off.

To take a broad view of these results obtained in controlled experimental conditions, it appears crucial to verify whether the same time-budget adjustments still apply at night, and whether they exist in threatened events in general and more specifically in natural systems where real predators could be encountered and where it is possible for birds to escape (see [1]).

## Materials and Methods

### Ethics statement

This work was performed under the governmental authorizations 67–99 and 67–285 delivered by the Préfecture du Bas-Rhin (Strasbourg, France) to conduct experiments on ducks and was approved by the Direction Départementale des Services Vétérinaires du Bas-Rhin (Strasbourg, France). The experiment complied with the “Principles of Animal Care” publication No. 86-23, revised 1985 of the National Institute of Health, and with current legislation (L87-848) on animal experimentation in France. After the study, ducks were released in the field under the control of the “Office National de la Chasse et de la Faune Sauvage” and with the authorization of the “Direction Départementale de l'Agriculture et de la Forêt du Bas-Rhin.”

### Animals and experimental conditions

The study was conducted over a three-year period as follows: the first year on 42 mallards split in three groups, the second year on 42 teals split in three groups and the last year on 28 tufted ducks split in two groups. Mallards were provided by the “La Canarderie de la Ronde” rearing centre (Cère la Ronde, France). Teals came from the Fauna Leroy rearing centre (Westvleteren, Belgium). Tufted ducks were obtained from the “Les Canards de Mormal” rearing centre (Jolimetz, France). Due to a supply problem, only two groups of tufted ducks were available for this study. Ducks were identified using individual color rings of Sellotape® which were loosely placed around one leg. Each group was composed of 14 individuals (7 females and 7 males) and was maintained in an outdoor tunnel-aviary of 100 m^2^ (20×5×2.5 m) subjected to ambient temperature and natural photoperiod. The aviaries were located near the laboratory and were protected against predators by an electric fence. The tunnels were 10–15 m from each other and were separated by opaque barriers to avoid visual contact between groups. All aviaries contained a 4 m^2^ pool (0.60 m depth) supplied with running water and placed in the same position in each tunnel, making the configuration identical for all groups. A balanced commercial food (Standard duck food 7751, Sanders Corporation) was provided *ad libitum* in feeders placed on 2×2 m covers to avoid food spillage. A richer protein food supplement (Teurlings premium duck food) was given *ad libitum* to tufted ducks because of their specific diet. All three species had at least one month's acclimation period for experimental conditions. The same aviaries were used for each species every year.

### Experimental procedure

#### Disturbance

We experimentally increased predation risk by increasing disturbance, therefore triggering typically evolved responses against natural predators [1], [30]. As a stressor, we used a radio-controlled car (E-Zilla FWD Hot-bodies™) to disturb the ducks by steering it towards the ducks at high speed until they took-off. This was the most efficient method to induce both simultaneous take-off of all birds in the group and predator attack-like response and ducks had never experience with such a stressor before precluding therefore any learning mechanism [30], [59], [60]. No duck was hurt by the car during these experiments. During disturbance phases, two experimenters (C.Z., M.B.) piloted the radio-controlled car from a corner of the aviary and noted the number of individuals taking off. Visual, thanks to the opaque barriers, and auditory cues during disturbance in one group did not modify the behaviour of ducks in the other groups.

Two groups of mallards and teals and one group of tufted ducks were disturbed over a one-week period four times during the wintering period between October and March, i.e. during non reproductive period. Approximately one and a half months was left between two successive disturbance sessions. In mallards and teals, animals of group 1 (G1) were disturbed twice daily for 15 minutes. In all three species, ducks of group 2 (G2) were disturbed four times per day for 15 minutes. Disturbance phases took place randomly between 8:00 and 11:00. In all three species, a control group (CG) was left undisturbed. During disturbance sessions, each aviary was monitored throughout the night with a night-view camera to ensure that ducks were not disturbed by any other external factors. Ducks of disturbed groups did not display any habituation to the radio-controlled car and took-off in reaction to the disturbance in all sessions.

#### Observations

In all three species, all individuals of each disturbed group were observed over the week before, during and after disturbance. The ducks of the control group were observed in the same way during this period. Each individual was observed with the focal animal sampling method [61] for 30 minutes every week. Observations took place between 11:30 and 17:00. The day and time for observation of individuals were determined by semi-randomization to avoid any risk of observing the same duck the same day of each week and at the same time in the four sessions. The observer (C.Z.) was located inside a small tent placed in identical position near to each of the three aviaries. Ducks were habituated to the observer's presence before the experiment began. To measure the time-budget of the individuals of the three groups, the durations of nine behaviours were recorded with a stopwatch. The behavioural units were divided into six different categories: (1) Foraging: taking food items in water, on the ground and from the feeders. (2) Vigilance behaviours: alert behaviours: duck raised its head or inclined the head with a stretched neck. “Motionless, awake, upright”: duck remained upright without moving and observed its surroundings. “Motionless, awake, lying down”: duck lay without moving and observed its surroundings. (3) Preening: cleaning the plumage with the bill or immersion of the head and the neck followed by a rapid raise of the body to sprinkle the back. (4) Sleeping: head placed on the back with bill under the scapulars or head bended with the bill placed on the plastron. (5) Peeking rate: number of peeks during sleeping period. A peek corresponds to short head raising and eye opening by birds during sleeping in order to scan their environment [40]. (6) Other behaviours: locomotion: walking and flying, swimming: locomotion in water. The three behaviours included in the last category account for less than 10% of the total diurnal time budget (9.0±0.8%) whatever the week and the species concerned. Moreover, these activities were often performed in association with other behaviours such as vigilance and foraging. These behaviours do not provide us with any new information and are not linked to the disturbance. They have therefore been excluded from the analysis.

### Statistical analysis

Differences in the time allocated to each behaviour class and variation in peeking rate in response to the disturbance were both modelled with a generalized linear mixed model (GLMM). In each model, session, group, sex and week (before, during and after the disturbance) were included as fixed factors. In order to take the repetition of the sessions and of the weeks within a session into account, these two factors were included as repeated factors with the week nested in the session. Our dataset is made of observations always related to a specific individual. Observations related to the same individual are positively-correlated. To account for pseudo-replication in the analysis, the repeated measurements within individuals were added as a random effect. A feature of mixed effects models used in the way we did is to take into consideration specific response of an individual (via the random effects) apart of the general response to a variable of interest (like the groups that are, in the present case, denoting the treatment and are included as fixed effect). Models were fitted with a gamma distribution for variations in behaviour duration and a Poisson distribution was used for changes in peeking rate using the GLIMMIX procedure (SAS 9.1.3). The INITGLM option was used to enable models to converge, using the estimates from a generalized linear model fit as the starting value for the generalized linear mixed model. The MSPL (Maximum Subject-specific Pseudo-Likelihood) technique was used as the estimation method. Tukey-Kramer multiple comparison adjustment was applied to obtain corrected p-value. Only significant effects were reported in the results section. Probability levels <0.05 were considered as significant. Mean duration values and peeking rates provided are given in seconds (s) ± SE.
